# Dysentery and Other Diseases in the Valley of Mill Creek, near Cincinnati, in the Summer of 1834

**Published:** 1834

**Authors:** G. Bailey

**Affiliations:** Cumminsville


### Art. II.—Dysentery and other Diseases.

*Observations on the Dysentery and othꞔr Diseases of the valley of Mill-creek, north of Cincinnati, in the spring and summer of* 1834. By G. Bailey, M. D.

When sickly seasons have passed away, it becomes the proper business of physicians to collect, and correctly set forth any remarkable medical facts they may have observed within the circles of their practice. Our intention, therefore, is to give a brief sketch of several circumstances connected with our practice in Cumminsville and the neighboring region, from the first of April last, up to this time. Brief it must be, for, not having any written notes to depend on, our memory must supply us with the requisite materials.

The circuit of our business, assuming Cumminsville as near the centre, embraces a space of from eight to ten miles diameter, every way.

Throughout the month of April and the first part of May, the principal disorders were affections of the lungs, in adults manifesting themselves under the various forms of pneumonia, pleuritis and catarrh, connected, at times, with biliary derangement; and in children generally assuming the form of congestive catarrhal fever, with soreness of the abdominal parietes, and marked disorder of the cerebral functions. Of adults, we lost none—of infants, a few. Our practice, we believe, was orthodox, and, therefore needs not be detailed. Looking back, however, upon our infantile cases, we have come to the firm conclusion, that our success would have been greater, if we had made a more liberal use of the lancet.

On the whole, this part of the season was healthy, and from the middle of May to that of June, no decided disease of any sort prevailed.

About the end of this term, we began to witness multiform derangements of the bowels—such as diarrhœa, bilious and watery, sick stomach, vomiting, cramp cholic and cholera morbus. Light colored watery diarrhœa has not been unfrequent during the whole season. It has occurred alone, or complicated with the different sorts of fever met with in this region. We have found it generally amenable to the timely administration of calomel and opium; in some instances, however, being compelled to give to the extent of ʒij. of the former combined with four or five grains of the latter. This combination sometimes failing, we have resorted, with excellent success, to the use of calomel in union with cayenne. Where no positive indications called for the administration of opium, we came gradually to prefer the use of this mixture, owing to the subsequent vexatious influence of opium in producing headache and obstinate torpor of the bowels. So obstinate has this been, in many instances, after the arrest of the diarrhœa, as to require heavy doses of the most active purgatives for its removal. A combination of calomel, aloes, and rhubarb, in equal parts, given to the extent of ʒij. and followed by four or five ounces of castor oil, has procured not more than two or three evacuations. The cayenne appearing to us almost as efficacious in arresting watery diarrhœa, never occasioned this inconvenience; and from its action being chiefly confined to the stomach and bowels, the head was generally unaffected by its exhibition.

In no case had we a diarrhœa of this kind to terminate in cholera. It is true, people were on their guard, and made timely application for medicine.

Cholera morbus occurred more frequently in the latter part of June, than at any other time. It was violent in its character, but always succumbed to our remedies. These were, after ascertaining that no crude matter remained in the stomach and intestinal canal, calomel and opium freely given—calomel and opium, separately given, in large or minute doses—calomel, camphor, and cayenne—sinapisms repeatedly applied, and injections of starch and laudanum. We found it necessary in all cases, to withhold drink of whatever kind, from the patients, except in the quantity of a tea-spoonful at a time. A solution of camphor in ether, as recommended by Dr. Eberle, we used frequently, with considerably good effect. Whether the white diarrhœal discharges were to be attributed to an inordinate indulgence in the vegetables of the season, or to a choleraic influence in the atmosphere, or to both conjointly, we will not discuss.

About the first of July, the dysentery began to prevail extensively, throughout the region of our practice, and continued, unabated in violence, until near the close of this month. Infants, children of from six to fourteen years of age, and adults, of from twenty to forty, were most largely affected. But the only fatal cases occurred amongst infants, owing to the impracticability of using with them the same efficient measures, as with older persons.

With regard to its localities, the bottom land, or lands along Mill creek, and the little streams emptying therein, were comparatively but little affected, while on the hills or highlands, towards Mount Pleasant, and about Blue Rock road, from seven to ten miles from this place, its attacks were frequent and severe.

The exciting cause of the disease, we believe, might, in every instance that came under our notice, be sought for in the presence of crudities in the intestinal canal, giving rise to irritation and inflammation. As to the circumstances that so generally predisposed the mucous membrane of the bowels to take on a dysenteric form of inflammation, under the influence of those exciting causes, we are silent. We have speculated, to be sure, but our speculations are so unsatisfactory to ourselves, that we cannot suppose they would satisfy others.

In some instances, the dysentery was of a highly inflammatory character, calling for rigorous antiphlogistic measures. Here a bold use of the lancet was required. A majority of the cases, however, did not present such a character. Amongst infants, in a few instances, the brain was seriously involved. Many times we had to contend with severe hepatic derangement, in which case emetics were never neglected. Sometimes, a well developed remittent fever was complicated with the complaint.

We endeavored, in our treatment, as is the habit of the profession, to moderate, when excessive, general excitement, to correct hepatic derangement, when present, to restore to a healthy condition, the always disordered cutaneous functions, and to lessen directly the violence of the local symptoms.

An excess of general excitement, demanding the use of the lancet, was of rare occurrence. Hepatic disturbance we generally rectified by the timely exhibition of one or two doses of ipecac., followed by scruple doses of calomel,or by calomel, ipecac., and opium. Our attention, however, was directed still more to the local affection and cutaneous derangement; for, correcting alone the disturbance of the biliary functions, and procuring bilious stools, were by no means, the infallible signs that a cure would follow.

To act upon the skin, therefore, and also on the bowels, opium, in watery solution, combined with ipecac, was our common remedy. We used it in the proportion recommended by Dr. Eberle, of a 1-4 grain opium to one or two grains ipecac. But a remedy of more specific influence on the local disorder, we found in the sugar of lead. In every variety and grade of the disease, we did not hesitate to use it in large doses, both by the mouth and in injections. According to the state of the liver or skin, or other circumstances, we gave it in combination with calomel and opium, or calomel and Dover's powder; with opium alone, or with opium and ipecac. Given in injection, we usually dissolved it in the watery solution of opium, occasionally with the addition of ipecacuan. For a child of eight or ten years old, our usual form was as follows:

Sacc. Saturni, ʒi.

Pulv. G. Opii., gr. vi.

Aquæ, ℥ iss.

One twelfth of this was used every two or three hours, or oftener, if the violence of the tormina and frequency of the discharges demanded. At the same time, to a child of the same age, two or three grains were administered every two or three hours, combined with some one or more of the articles noted above.

Castor oil, as a laxative appeared to us, not unfrequently to aggravate the disease. In such cases, we often prescribed calomel alone, in doses of 10 or 20 grains to children, as an efficient and gentle purgative. In dysentery it is said that the local irritation gives rise to spasmodic constrictions of the intestinal tube. This being the case, it is easy to understand why castor oil, acting, as we suppose, by quickening and energizing the peristaltic motion, not unfrequently increases greatly the tormina and tenesmus. We know of no reason, therefore, why some of the saline laxatives, that operate chiefly by stimulating the intestinal exhalents to an increased discharge of fluid, thereby unloading the engorged blood vessels, about the abdominal canal, should not be more beneficial than castor oil. Hence, we perceive some philosophy in the assertion of Dr. Cheyne, that ½ oz. of cream of tartar, finely levigated, and given every four or six hours, has restored patients to health, who could not, he believes, have otherwise recovered. My colleague speaks well of the strychnine, in this complaint. In two cases, after all the usual remedies had failed, he succeeded entirely with this article. They were both children, one two, and the other three years old. One twentieth of a grain of strychnine was administered three times, daily, for three or four days, until a decided abatement of all the symptoms took place.

As external applications, we made constant use of sinapisms and fomentations, never resorting to epispastics. The warm bath was also employed with advantage.

From the end of July to this date, we have had occasional cases of Dysentery, but much milder in character, and more readily amenable to medicine. A few doses of calomel and Dover's powder, together with the application of sinapisms, are the chief agents now requisite to subdue the disease.

During the last six weeks our chief complaints have been remittent and common continued fevers, connected generally with hepatic derangement. Their characters have been various. In some cases the onset has been violent, and the fever of a high grade; the sanguiferous system being chiefly involved. The attack in other cases has been gradual, the whole secretory system then appearing disordered, and not unfrequently the nervous. Hence we have torpid liver, skin, bowels, and kidneys, while perhaps the pulse is scarcely indicative of disease. Such cases are dangerous, require peculiarly discreet and efficient treatment, and are apt to be long and obstinate in their course. Bloodletting is of doubtful efficacy, except in the earliest period of the disease: but we cannot say, that even then we have derived any decided benefit from it. To bring the system under mercurial influence, and thus to destroy or supplant the train of morbid actions so firmly established, has been a prime object with us. When this could be done, we felt confident of effecting a cure. It is in place here to remark, that although in such instances, the greatest difficulty has been experienced in producing ptyalism; yet, in most other cases, we have noticed a peculiarly strong predisposition to the mercurial sore mouth—so much so, that whenever we gave calomel, we had to guard particularly against ptyalism.

We have had cases of fever, associated with most obstinate torpor of the bowels, and others, accompanied with profuse and watery diarrhœa from the commencement; these requiring the most powerful anti-irritants, and those surprising doses of the most active cathartics.

My colleague has furnished me with the particulars of a case which he attended, wherein the immense quantities of medicine necessary to move the bowels, may seem almost incredible. The patient, a middle aged man, was affected with a low fever, attended by oppression at the stomach, torpor of the bowels, and a thickly furred tongue. Thirty grains of calomel were administered, followed in four hours afterwards by four pills, the whole containing of calomel, aloes, and rhubarb, each 8 grains. These were followed at short intervals, by 8 more of the same kind. No operation resulted, and on the next day, sixteen of the same pills were given, at short intervals, but without any effect. On the succeeding day, ʒis. of cal. aloes. and jalap, in equal parts, were directed to be taken—one half immediately, the other in the course of four hours, if necessary. Both were taken, but the bowels remained unmoved. ʒiij. of the same compound were left on the following morning, to be divided in two equal portions, and taken within a few hours of each other, if requisite. The patient sent word at the close of the day, that all the medicine was taken, and not the slightest operation procured. 2 oz. of senna with 4 oz. epsom salts, were immediately sent over. The directions were, to make a pint of tea with one-half the quantity sent, and to take one-fourth of it every one or two hours. If this failed, the other half was then to be used in like manner. The directions were observed. Nearly the whole quantity of senna and salts were taken, and four bilious operations were the result. Constant purgation, with large doses of active cathartics, was persevered in, and the man got well.

As it regards Cholera, we are happy in saying we have little to report. From the first of April to this date, we have had but one case, within the usual range of our practice. That occurred on the hills towards Mount Pleasant, in the month of July, and was fatal. It might not have been so; for, the plainest indications which vomiting purging, and excessive pain can give, were disregarded for hours, before medical aid was called. Two cases in one family, about eight miles north-west of this place, and three cases in another family, twelve miles north of it, all of an exceedingly violent character, were attended by my colleague, Dr. Mount.

Intermittent fevers, of rare occurrence for some time past, are beginning to be rather more frequent. The health of this region, however, is generally excellent, and that it may continue so, our vocation, as some slanderously affirm, does not prevent us from sincerely wishing.

*Cumminsville, September* 16, 1834.

We beg leave respectfully to solicit of our brethren, every where in the West, accounts of the late Epidemics, some notices of which are given in the foregoing articles. They should be brief and condensed, omitting, as far as possible, all matters which are universally present in those forms of disease. Ed.

